# JOBCR has new Editor - in - Chief

**DOI:** 10.1016/j.jobcr.2023.10.002

**Published:** 2023-10-20

**Authors:** Vidya Rattan

**Affiliations:** Oral Health Sciences Centre, Postgraduate Institute of Medical Education and Research, Chandigarh, India

I have taken over the charge as new Editor in Chief since August 2023. I embark upon this new journey with great pride, enthusiasm and sense of responsibility. I am a full-time academician working as Professor of Oral & Maxillofacial Surgery at Postgraduate Institute of Medical Education and Research, Chandigarh, involved in patient care, teaching and research for the last 30 years.

The JOBCR has become a reputed journal over a short span of 10 years with nurturing, dedication and hard work of Dr Divya Mehrotra. Unfortunately, sudden demise of Dr Divya has left a great void which is difficult to fill. Lately there has been certain issues with the decision-making process because of lack of her guidance, which I definitely intend to sort out.

My efforts will be to take this journal to greater heights by attracting and publishing quality research, thereby improving citation score and ranking of journal. The scope of this journal is quite vast therefore, to bring more representation I intend to bring new set of section editors, editorial team members and reviewers onboard to cover diverse topics. I also intend to start regular editorials, invited reviews, commentary for important articles and publish special issues on current topics. The aim is not only to improve impact factor of the journal but to have an impact in the lives of our patients by publishing latest clinical research, new innovations and cutting-edge research.

There is one more major change which has happened to the journal i.e., it has become gold open access. There are advantages of open access technology in the form of free access for wider unrestricted dissemination. Now the published articles can be accessed instantly without any charges by everyone across the world. There are also special reduced publication charges for low-income countries so that the expense on publishing an article should not become prohibitive.

Rapid turnaround time, efficiency and transparency will be my work mantras. This all cannot be achieved without the active support of editorial team members. I seek active support from all the members to achieve these objectives and to make the journal number one destination for publishing quality research.

